# Effect of Dill (*Anethum graveolens*) Oil on Pruritus and Quality of Life of Hemodialysis Patients: A Randomized Double-Blind Three-Arm Controlled Trial

**DOI:** 10.1155/2024/3077603

**Published:** 2024-01-12

**Authors:** Zeinab Shaki, Farzaneh Ghaffari, Fatemeh Alijaniha, Mohammad Kamalinejad, Anoshiravan Kazemnejad, Babak Daneshfard, Mohsen Naseri, Mohammad Reza Heidari

**Affiliations:** ^1^Department of Nursing, Faculty of Nursing and Midwifery, Shahed University, Tehran, Iran; ^2^Traditional Medicine Clinical Trial Research Center, Shahed University, Tehran, Iran; ^3^School of Traditional Medicine, Shahid Beheshti University of Medical Sciences, Tehran, Iran; ^4^School of Persian Medicine, Shahed University, Tehran, Iran; ^5^School of Pharmacy, Shahid Beheshti University of Medical Sciences, Tehran, Iran; ^6^Department of Biostatistics, Faculty of Medical Sciences, Tarbiat Modares University, Tehran, Iran; ^7^Chronic Respiratory Diseases Research Center, National Research Institute of Tuberculosis and Lung Diseases (NRITLD), Shahid Beheshti University of Medical Sciences, Tehran, Iran; ^8^Persian Medicine Network (PMN), Universal Scientific Education and Research Network (USERN), Tehran, Iran; ^9^Hikmat, Islamic and Traditional Medicine Department, The Academy of Medical Sciences, Tehran, Iran

## Abstract

**Introduction:**

*Anethum graveolens* (AG), commonly known as dill, is a plant from the Apiaceae family that has been traditionally used as a skin softener and purifying agent in Persian medicine. In a previous study conducted on male rats, dill was found to have anti-inflammatory effects. The current study aimed to examine the efficacy of topical application of *Anethum graveolens* oil on pruritus severity, skin dryness, sleep quality, and quality of life in patients undergoing hemodialysis.

**Methods:**

In the current clinical trial, the participants were randomly assigned to one of these groups: topical AG preparation, sesame oil, or a control group receiving no treatment. The study was double-blind and placebo-controlled. The topical treatment was applied twice a day for one month to areas of the skin affected by pruritus. The outcome measures included the severity of skin dryness, the Pittsburgh Sleep Quality Index, Duo's Uremic Pruritus Severity Scale, and the Itchy QoL questionnaire.

**Results:**

This study involved 106 hemodialysis patients, and after intervention for one month, the medication group had a significantly lower mean score of sleep quality (3.24 ± 2.41) than the placebo (4.54 ± 3.11) and control (5.05 ± 3.21) groups (*P*=0.032). The mean change in pruritus severity was significantly greater in the medication group (−17.28 ± 8.938) than the placebo (−5.91 ± 5.398) and control (−3.43 ± 3.228) groups (*P* < 0.001). Moreover, a significant difference was observed in the mean changes in quality of life between the medication, placebo, and control groups, with values of −14.88 ± 7.89, −5.34 ± 4.50, and −1.92 ± 2.86, respectively (*P* < 0.001). Furthermore, both the medication and placebo groups showed improvement in skin dryness compared to the control group having the values of −1.65 ± 0.91, −1.11 ± 0.79, and −0.38 ± 0.54, respectively (*P* < 0.001).

**Conclusion:**

Topical *Anethum graveolens* preparation significantly improved the sleep quality and quality of life and reduced skin pruritus and dryness in hemodialysis patients. It could be considered as a simple therapeutic modality to control pruritus in hemodialysis patients. This trial is registered with IRCT2017022032671N1.

## 1. Introduction

Uremic pruritus is a commonly occurring symptom that affects around 52% of the population in Iran [1]. The exact cause of uremic pruritus is unknown; however, various factors, such as skin dryness, inflammatory immune responses, pruritus cytokines, secondary hyperparathyroidism, and uremic neuropathy, are believed to contribute to its development [2–4]. Skin dryness is closely linked to pruritus severity in patients undergoing hemodialysis, and its incidence varies from 76.2% to 83% across different regions of the world, with an estimated prevalence of 51% in Iran [1, 3]. Uremic pruritus can lead to sleep disorders, negatively affecting the quality of life [4, 5]. Additionally, it may increase the likelihood of depression and mortality [6, 7].

There are various treatments, both pharmacological and nonpharmacological, that have been suggested beneficial for improving the sleep quality in patients with uremic pruritus. Some medications, including gabapentin, pregabalin, ketotifen, nalbuphine, and montelukast, are effective in controlling pruritus [8]. Topical products like moisturizing creams and baby oil can alleviate pruritus caused by skin dryness [9]. Other nonpharmacological remedies consist of enhancing blood flow in the hemodialysis machines, as well as utilizing acupuncture, acupressure, aromatherapy, and narrowband ultraviolet phototherapy [10, 11]. Additionally, modifying nutritional status, vitamin D levels, and employing cold hemodialysis are among other helpful strategies [12, 13].

The utilization of traditional medicine can be considered as a novel mechanism for managing various diseases in an evidence-based integrative approach [14, 15]. Persian medicine (PM), which has been practiced for thousands of years, provides numerous effective treatments for various ailments [16, 17]. PM encompasses various therapeutic modalities, including dietary, pharmaceutical, and manual interventions. Herbal medicine has been particularly emphasized [18, 19].

Pruritus is a prevalent complication that affects many patients before starting dialysis, negatively impacting their quality of life and sleep [20]. With the increasing popularity of complementary and alternative medicine (CAM), patients have begun seeking alternative options [21]. Some CAM treatments, including topical capsaicin, acupuncture, and acupressure, have been shown to be effective in treating uremic pruritus [22]. Additionally, phytotherapy is a popular CAM treatment for pruritus management [23], and various topical oils, such as violet, clove, peppermint, sweet almond, baby oil, and ostrich oil, have been found to be beneficial in managing pruritus symptoms [9, 24–34]. Nutritional interventions and hatha yoga exercises may also improve sleep quality in hemodialysis patients [12, 33].

It possesses various medicinal properties, including analgesic, antioxidant, and anti-inflammatory effects [34]. Pruritus has been accounted as a type of pain in Avicenna's masterpiece, Canon of Medicine. In the second book of Canon, which is dedicated to Materia Medica, dill oil has been introduced as a strong skin softener with analgesic and hypnotic properties [35]. Dill oil has also been used as a relieving agent for neural pains, arthralgia, arthritis, otalgia, and anal ulcers [36].

AG essential oil is composed of various major components, such as carvone, limonene, dihydrocarvone, carvacrol, p-cymene, *α*-phellandrene, and dill apiole [37]. Phenol and flavonoids, which are potent radical scavengers, have recently been considered as possible therapeutics against free radical mediated diseases, such as inflammatory disorders [38, 39]. Boiled AG seeds have been found to be helpful in the labor process [40, 41] and recommended for sleep disorders [42]. In our previous animal study, AG oil was found to have significant anti-inflammatory effects on formalin-induced inflammation in male rats [43]. This clinical trial was conducted to study the impact of AG oil on pruritus and quality of life factors in hemodialysis patients. This study is the first of its kind and was carried out due to the belief that inflammation contributes significantly to pruritus development.

## 2. Methods

### 2.1. Study Design

Adopting a randomized, double-blind, and placebo-controlled design, the current trial was conducted on hemodialysis patients in hemodialysis centers of Golestan University of Medical Sciences in Golestan province, Iran. Following the CONSORT guideline, participants were allocated to three groups of medication (AG oil), placebo (sesame oil), and control (no medication) with equal allocation ratio. The medication and placebo were completely similar in appearance and their contents were unknown to both patients and the investigator.

## 3. Participants

### 3.1. Inclusion and Exclusion Criteria

The criteria for including participants in the study were as follows: being 18 years or older, having undergone hemodialysis in the last three months, experiencing at least three episodes of pruritus lasting five minutes or more in the last two weeks, and scoring a minimum of 3 on the Visual Analogue Scale (VAS) for pruritus. Participants were excluded if they had liver problems, open wounds, cellulitis, infections, deep vein thrombosis, epilepsy, bleeding disorders, paraplegia, a pacemaker, hearing or speech difficulties, had undergone kidney transplantation, or had allergies.

### 3.2. Intervention

During the study, patients were instructed to apply 2.5 g of either AG or sesame oil topically to the skin twice a day for one month. They were provided with instructions on avoiding contact with eyes and washing their hands with cold water after each use and cautioned against using the oils on areas with open wounds.

### 3.3. Outcomes

The outcome measures comprised the severity of skin dryness, the Pittsburgh Sleep Quality Index, Duo's Uremic Pruritus Severity Scale, and the Itchy QoL questionnaire. Before and after the intervention, the blood samples were collected from participants and sent to the laboratory for analysis. The tests included measuring levels of phosphorus, calcium, alkaline phosphatase, creatinine, blood urea nitrogen, hemoglobin, hematocrit, and platelet count, as well as the levels of C-reactive protein (CRP) and parathormone. Participants were randomly assigned to groups receiving either the medication (AG oil) or placebo. The control group received routine therapeutic measures.

The skin sensitivity was first checked with the content of the package before the intervention. First, a hand without fistula was washed with soap and water and dried. At the beginning of hemodialysis, a small amount of the content of package was applied to the skin. During the hemodialysis, the site was observed for any type of reaction. The patient was then asked to report any kind of reaction during the study period. Ensuring that no complication occurred in the first session, the patient was trained for the correct manner of taking the topical oil at the presence of a physician who was a member of the research team. The patients were also asked to share any question or problem immediately via phone numbers that were given to them and their family members.

After the end of a one-month period, the tests were repeated and questionnaires were completed under the supervision of one of the researchers.

### 3.4. Sample Size

Thirty-four individuals were selected as the sample size that was calculated using the following sample size formula by taking into account the proportion of the 10% dropout according to a similar study [27]:

(1) $$\begin{array}{c} n=\frac{{\left(Z_{1-\alpha /2}+Z_{1-\beta }\right)}^{2}\left(S_{1}^{2}+S_{2}^{2}\right)}{{\left(\bar{X_{1}}-\bar{X_{2}}\right)}^{2}},\\ n=\frac{{\left(1.96+0.84\right)}^{2}\left({\left(2.9\right)}^{2}+{\left(5.5\right)}^{2}\right)}{3^{2}}=33.9\approx 34,\\ 34+34\left(0.1\right)\approx 37. \end{array}$$

#### 3.4.1. Randomisation

In this research, the samples were selected by the available sampling method. In order to generate the random allocation sequence, the samples were randomly assigned. Considering the inclusion criteria, the participants were chosen using the convenience sampling method and then divided into three groups using a computer-generated block randomization method. The block size was four. Next, 111 patients undergoing hemodialysis were selected and randomly assigned to three groups: drug, placebo, and control (without intervention).

#### 3.4.2. Blinding

The packages containing either the AG oil or the placebo (sesame oil) were identical in color, size, and shape and were randomly labeled with codes that remained confidential by an independent individual until the trial ended. During the study, a nurse who was unaware of the allocation of the packages administered the medication (AG oil) and placebo packages to the patients.

#### 3.4.3. Data Collection Instruments

Demographic and clinical characteristics of patients as well as laboratory parameters, including calcium, phosphorus, parathormone, CRP, blood urea nitrogen, creatinine, hemoglobin, hematocrit, alkaline phosphatase, and platelets were recorded.

We used a grading method to evaluate skin dryness which had been explained in a previous study [44]. According to this method, score 0 indicates the absence of skin dryness; score 1: mild skin pruritus (involving legs); score 2: moderate skin dryness (involving all organs); and score 3: severe skin dryness (involving the whole body).

The Pittsburgh Sleep Quality Index (PSQI-P) in Persian version was utilized to evaluate the patients' quality of sleep [45]. It includes different dimensions of sleep disorders questioning about problem in getting to sleep, during the sleep, and in waking up. Four questions are considered for each type of sleep disorder. The score of 0 or 1 is considered for each question depending on the existence of the problem. The mean score of all four questions is named as PSQI-P. Patients with the PSQI-P less than 4 are assumed to have a good sleep, but those with PSQI-P of 4 or greater are considered to have a sleep disorder.

In addition, Duo's uremic pruritus scale was applied to the pruritus severity assessment. This questionnaire investigates the frequency, severity, and dispersion of pruritus and the resulting sleep disorder. Pruritus severity is evaluated by a score range of 0–48. A higher score indicates higher severity of the problems [46].

The ItchyQoL questionnaire, which has 26 questions evaluating symptoms, functions, and feelings of patients with pruritus, was used to assess the clinical status of the participants in terms of their quality of life. The answers were scored on a scale of 1 to 5. The scores of each participant were calculated on each axis and the total score. A previous study had confirmed the reliability and validity of the Persian version of the questionnaire. There were no modifications made to the trial outcomes [47].

#### 3.4.4. Validity and Reliability of Instruments

The Visual Analogue Scale (VAS) tool has already been used for itching severity in clinical trials in Iran and around the world [9, 48, 49]. It is a visual, numerical single-item tool that can be easily translated and shown to the patients. The validity of the tool for measuring skin dryness was proven in a study by Akhyani et al. [44]. In order to check the sleep quality of patients, the postsleep inventory (PSI) questionnaire, designed by Webb et al. [50], has been used by researchers to evaluate the subjective responses to the last period of sleep [51, 52]. Janabi et al. translated, validated, and used the modified version of PSI (modified postsleep inventory) questionnaire to investigate the relationship between C-reactive protein serum level and sleep disorders in chronic hemodialysis patients [53].

The itch intensity questionnaire used in this research was originally designed by Duo [46] and then modified by Mettang et al. [54]. It was then modeled by Yosipovitch et al.'s [55] questionnaire and was first translated into Persian before being used by Panahi et al. In the current study, the researchers adopted and used this Persian version by Panahi et al. [49].

The questionnaire for the quality of life of patients suffering from itching (ItchyQoL) is related to the research of Desai et al. in 2008 [47]. Tari et al. translated the ItchyQoL questionnaire and checked its reliability and validity [56]. To check the reliability and validity of the Persian translation of the ItchyQoL questionnaire, it was first translated into Persian by two bilingual experts and then back-translated into English by two other bilingual experts. Finally, by matching the original and translated versions and revising the questions of the Persian version of the questionnaire, it was presented to the patients. The questionnaire was filled in by 44 patients with pruritic skin disease in two occasions with an interval of 72 hours. Then, the answers were statistically analyzed to check the reliability and structural validity of the questionnaire. The reliability of the questionnaire was determined by calculating the internal correlation coefficient (Cronbach's alpha correlation coefficient) and the test-retest method.

### 3.5. Statistical Analysis

The analysis of the data was performed using the Statistical Package for the Social Sciences software (version 18.0). Using the one-sample Kolmogorov–Smirnov test, the normality of continuous variables was checked. The chi-square as well as Fisher's exact tests were also used for nominal variables, while the Kruskal–Wallis and Mann–Whitney tests were performed for categorical variables. One-way ANOVA and Tukey's post hoc test were used in the case of continuous variables with a normal distribution. Statistical significance was considered as a *p* value of less than 0.05.

### 3.6. Herbal Medicine Preparation

The AG fresh plant was procured from a local market in Tehran and confirmed as authentic at the Herbarium of the Faculty of Pharmacy, Shahid Beheshti University of Medical Sciences, Tehran, Iran, with the voucher number 1702. To prepare the topical oil, 40 ml juice was extracted from 100 grams of AG fresh aerial parts, which was then mixed with sesame oil obtained from the Saman Company. The mixture was boiled at a gentle temperature, so that all existing water in the mixture evaporated within 2 hours and the oil phase was remained. In the end, 44 g of AG oil in sesame oil base was obtained. The mentioned preparation as “AG oil” was used in the medication group while the sesame oil was used in the placebo group.

### 3.7. Phytochemical Study of AG Oil

#### 3.7.1. Total Fatty Acid Content

The AG oil fatty acid content was determined using the gas chromatography and mass spectrometry (GC-MS) method. In brief, trans-methylation of fatty acids was carried out by accurately weighing 500 mg of AG oil into a screw-capped tube, to which 3 mL of heptane was added and mixed until the oil was completely dissolved. Methylation was then performed using a 2 N methanolic potassium hydroxide solution (2 mL). The tube was shaken for 30 seconds, and the aqueous and organic layers were allowed to separate. A 2 *μ*L sample from the top layer (heptane) was then injected into the gas chromatograph for analysis.

A gas chromatograph which was coupled with a mass spectrometer, specifically the Agilent 6890 system, was employed in the analysis. Separation of the compound mixture was carried out on a BPX5 column (30 m × 0.25 mm × 0.25 mm film thickness) using a program of temperature that ramped from 50 (held for 1 minute) to 133°C (held for 0.2 minutes) at a rate of 3°C per minute. The temperature of the injector was set at 290°C, and injection was performed in the splitless mode. Helium was used as the carrier gas, at a flow rate of 1 ml/min.

### 3.8. Determination of Phenol and Flavonoid Contents

Folin–Ciocalteu reagent was used to determine the total phenolic content of AG oil (based on gallic acid), following a method previously described (36, 60). Gallic acid (Sigma) was used as the standard. To extract the sample of AG oil, methanol was used. Then, 5 ml of reagent (Folin–Ciocalteu reagent diluted with distilled water 1 : 10) as well as 4 ml of Na_2_CO_3_ 1M were added to both the sample and standard solutions. The total content of phenolic compounds was determined by colorimetry at 765 nm after 15 minutes. By measuring the absorption of gallic acid solution at 0.0000, 50.0000, 100.000, 200.000, and 250.000 mg/ml concentrations in methanol:water (50 : 50 v/v), a standard curve was prepared. The tests were repeated three times, and the mean value was presented as gallic acid equivalent (mg/g of AG oil).

The total flavonoid content of AG was measured using the method described by Beketov et al. [57]. A mixture of 5 ml of 2% aluminum trichloride in methanol and 5 ml of AG oil was centrifuged at 4000 rpm for 8 minutes. The upper clear phase was separated, and the total flavonoid content was determined by calorimetry at 415 nm, using a blank sample of AG oil and methanol. The standard curve was prepared using a catechin solution (Sigma, Aldrich), with concentrations of 0.0000, 5.0000, 20.000, 50.000, and 80.000 mg/l. After three measurements, the mean values of the total flavonoid content were reported as mg of catechin equivalent per gram of AG oil.

## 4. Results

### 4.1. Participant Flow

**Figure 1:**
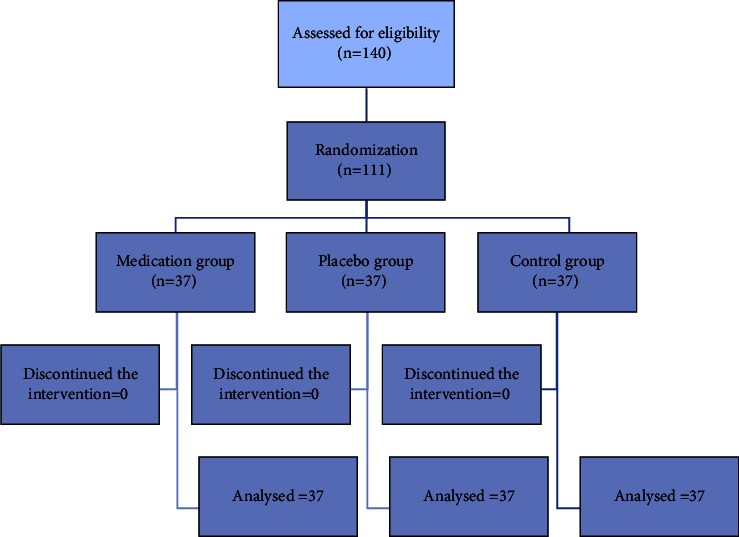
CONSORT diagram of the trial.

All 111 initial participants completed the study during 2016-2017. The trial's CONSORT flow diagram is presented in Figure 1.

### 4.2. Patient Characteristics

Tables 1 and 2 display the demographic characteristics of the patients and the results of laboratory tests. The data indicate that no significant differences between the groups regarding these factors were observed, except for the underlying cause of kidney failure.

The result of the Kruskal–Wallis test showed that before the intervention, there were some differences between the mean creatinine values. There were statistically significant differences between the three groups. There was a significant difference by entering the creatinine variable into the ANOVA model as the covariate and the ANCOVA was analyzed, and it was observed that the difference in creatinine in the groups did not have any effect on the significance of the differences between the groups for the main purposes.

### 4.3. Pruritus Severity

Prior to the intervention, significant differences were observed among the mean pruritus severity scores of the placebo, medication, and control groups (Table 3). Therefore, the ANOVA test was employed to compare the changes of pruritus severity between the three groups, which indicated higher decrease in the pruritus severity score in the AG group than the other groups during the study (*P* < 0.001).

### 4.4. Skin Dryness

Skin dryness degree was significantly different before the intervention in the medication, placebo, and control groups (*P*=0.036). However, ANOVA test showed that mean changes of skin dryness scores were significantly different after the intervention among the groups (*P* < 0.001) (Table 4).

### 4.5. Quality of Life

The study found that the quality of life at the beginning of the study was different between groups (*P*=0.018). After intervention, the ANOVA test was used for comparing the score changes between groups and it showed a significant difference (*P* < 0.001) which is presented in Table 5. The post hoc test revealed a significant difference between medication and placebo groups (*P* < 0.001), medication and control groups (*P* < 0.001), and placebo and control groups (*P*=0.024) in terms of mean changes in the quality-of-life scores. The participants did not report any serious side effects.

### 4.6. Sleep Quality

Table 6 displays the results of the study regarding the mean scores of sleep quality in the three groups before and after the intervention. The results of the analysis showed that there was no significant difference in the mean scores of sleep quality among the groups before the intervention (*P*=0.687). However, after the intervention, a significant difference was observed among the groups (*P*=0.032). At baseline, the PSQI-P score for all groups was above 4, indicating sleep disorders. Nevertheless, after the intervention, only the medication group demonstrated a PSQI-P score below 4, indicating improved sleep quality.

### 4.7. Phytochemical Study of AG Oil

The values for fatty acid contents of AG oil are shown in Table 7. Moreover, total phenol and flavonoid contents of AG oil were determined as 0.081 ± 0.002 mg gallic acid/g and 0.12 ± 0.04 mg catechin/g, respectively.

### 4.8. Harms

No adverse effects or hypersensitivity were observed in the study groups of the current clinical trial.

## 5. Discussion

The clinical treatment of uremic pruritus starts with changing the dialysis method, and improving the quality of dialysis can reduce the prevalence and severity of ESRD pruritus, but these measures have a high cost and treatment complexity, so their application is limited [58, 59]. A better quality of life can be achieved for these patients through planning and implementing nursing interventions to increase the adequacy of dialysis and to improve some parameters [60].

The findings of this study reveal that the medication group experienced significant improvement in pruritus, skin dryness, sleep quality, and overall quality of life compared to the other groups. No adverse reactions or hypersensitivity were observed following the use of AG topical preparation.

Essential oils as secondary metabolites of plants have been reported to have different functions, such as antimutagenic, antioxidant, antibacterial, antiviral, antifungal, and anti-inflammatory properties [61]. For example, a randomized controlled clinical trial study demonstrated that topical use of traditional formulation of chamomile oil can decrease the analgesic requests of patients with knee osteoarthritis [62]. In another study, Esmaeili et al. demonstrated that cinnamon and clove essential oil nanogels could be regarded as analgesic drugs for preventing the inflammation and nociception in rats [63]. A recent randomized double-blind, controlled trial investigation disclosed that supplementation with *Nigella sativa* oil could decrease oxidative stress, inflammation, and blood sugar in diabetic patients undergoing hemodialysis [64]. In the current study, sesame oil was employed as a common oily vehicle for medicinal oils, which has been recommended for formulation in traditional Persian medicine. Moreover, sesame oil has been reported to have antioxidant and anti-inflammatory properties [62].

Given that pruritus affects a high percentage (20–80%) of hemodialysis patients and significantly impacts their quality of life, these results are of significant importance [65].

AG could probably reduce itching through its anti-inflammatory effects; thus, the patients have experienced a better sleep quality in addition to the improved quality of life. The findings of the present study are in line with the results of our previous research that stressed the anti-inflammatory effect of *Anethum graveolens* oil [43]. Also, given the antidysmenorrhea effects in a study by Mohammadinia et al., the anti-inflammatory effects of the AG extract can be taken into consideration [66].

The results of the current study confirm that our intervention improved the pruritus severity in patients compared with the control and placebo groups. In a cross-sectional clinical trial, massage therapy was carried out with/without aromatic oil. Analysis of research data indicated that the massage with or without aromatic oil significantly relieves itching [67]. Accordingly, it seems that a portion of our intervention efficacy might be due to the massage effect.

In another double-blind randomized trial, the effect of capsaicin on the uremic pruritus was examined. In this double-blind cross-over clinical trial on 34 hemodialysis patients suffering from uremic pruritus, the effect of capsaicin compared to placebo was investigated. The results showed that the effect of capsaicin and placebo on reducing the severity of itching was the same; so, the researchers recommended that considering that the placebo contained a moisturizer, patients should use topical moisturizers to reduce their itching. It was found that capsaicin reduces the pruritus severity similar to the placebo. They showed that the use of capsaicin 0.03 is effective in improving and relieving itching [27]. Nevertheless, patients reported burning sensation after using capsaicin while no adverse effect was reported in the current study.

The study found no significant difference in mean changes in skin dryness between the medication and placebo groups. However, differences which were significant were observed between the placebo and control groups, as well as differences between the medication and control groups, in terms of mean changes in skin dryness. This suggests that both the medication and placebo groups experienced improvements in skin dryness compared to the control group, potentially due to the presence of sesame oil.

Improving the effect of AG oil on pruritus and skin dryness can well explain its positive effect on the sleep quality and quality of life of the patients regardless of its traditional use for the sleep disorders [68].

Small sample size, short duration of follow up, and subjective outcome measures may be regarded as the limitation of the present research, which should be considered in future studies.

## 6. Conclusion

It can be concluded that *Anethum graveolens* oil (on the basis of sesame oil) can effectively reduce the pruritus and skin dryness of the hemodialysis patients and thus improve their sleep quality and quality of life.

## Figures and Tables

**Table 1:** Characteristics related to demographics and clinical information of the participants.

Variable		Medication (*N* = 37)	Placebo (*N* = 37)	Control (*N* = 37)	*P* value
Age (years) (mean ± SD)		62.86 ± 11.31	54.14 ± 13.651	58.08 ± 11.987	0.11

Gender (*N*, %)	Female	20 (54.1)	22 (59.5)	24 (64.9)	0.641
	Male	17 (45.9)	15 (40.5)	13(35.1)	

Marital status (*N*, %)	Single	0 (0)	2 (5.4)	1 (2.7)	0.361
	Married	37 (100)	35 (94.6)	36 (97.3)	

Education level (*N*, %)	Illiterate	17 (45.9)	17 (45.9)	18 (48.6)	0.467
	Literacy for reading and writing	4 (10.8)	0 (0)	4 (10.8)	
	Under high school diploma	11 (29.7)	16 (43.2)	8 (21.6)	
	High school diploma and higher	5 (13.5)	4 (10.8)	7 (18.9)	

Body mass index (BMI) (kg/m^2^)(mean ± SD)		26.35 ± 4.103	27.42 ± 5.375	26.38 ± 3.500	0.494

History of smoking (*N*, %)	Yes	2 (5.4)	4 (10.8)	0 (0)	0.123
	No	35 (94.6)	33 (89.2)	37 (100)	

Cause of kidney failure (*N*, %)	Diabetes	12 (32.4)	7 (18.9)	9 (24.3)	0.087
	Hypertension	6 (16.2)	16 (43.2)	8 (21.6)	
	Diabetes and hypertension	9 (24.3)	10(27.0)	14 (37.8)	
	Others^*∗*^	10 (27.0)	4 (10.8)	6 (16.2)	
		5 (13.5)	4 (10.8)	7 (18.9)	

History of hemodialysis (month) (mean ± SD)		46.14 ± 32.628	43.86 ± 31.855	44.41 ± 39.030	0.780

Adequacy of dialysis (Kt/*V*^*∗∗*^) (mean ± SD)		1.32 ± 0.245	1.25 ± 0.183	1.28 ± 0.199	0.355

Duration of pruritus (week) (mean ± SD)		58.03 ± 69.191	66.24 ± 59.124	54.84 ± 51.006	0.395

^*∗*^Polycystic kidney disease, hydronephrosis, glomerulonephritis, or unknown. ^*∗∗*^Kt/*V*_urea_ (“*K*” is dialyzer urea clearance, “*t*” is total dialysis session time, and “*V*” is volume of distribution of urea).

**Table 2:** Laboratory test results of the patients.

Variables/normal range (unit)		Medication group, *N* = 37 (mean ± SD)	Placebo group, *N* = 37 (mean ± SD)	Control group, *N* = 37 (mean ± SD)	*P* value
Calcium/8.5–11.5 (mg/dl)	Before the intervention	8.70 ± 0.532	8.68 ± 0.582	8.85 ± 0.769	0.355
	After the intervention	8.92 ± 0.755	8.80 ± 0.894	8.74 ± 0.934	0.672

Phosphorus/2.5–5.5 (mg/dl)	Before the intervention	4.86 ± 0.854	5.05 ± 0.748	5.16 ± 0.918	0.304
	After the intervention	4.84 ± 0.904	4.85 ± 0.867	5.45 ± 1.200	0.015

BUN/8–23 (mg/dl)	Before the intervention	92.03 ± 44.366	106.70 ± 39.131	122.95 ± 49.660	0.014
	After the intervention	95.06 ± 52.664	107.17 ± 46.190	109.78 ± 51.638	0.429

Creatinine/0.5–1.5 (mg/dl)	Before the intervention	11.38 ± 18.293	12.77 ± 16.935	8.78 ± 1.707	0.010
	After the intervention	8.66 ± 2.170	9.59 ± 2.179	8.58 ± 2.303	0.111

Hemoglobin/12–17 (mg/dl)	Before the intervention	11.21 ± 1.730	10.59 ± 1.499	10.89 ± 1.676	0.271
	After the intervention	11.23 ± 1.918	11.02 ± 1.725	10.95 ± 1.609	0.792

Hematocrit/33–51 (%)	Before the intervention	34.67 ± 4.814	32.44 ± 5.023	33.98 ± 5.001	0.146
	After the intervention	35.27 ± 5.429	33.94 ± 4.311	34.56 ± 5.074	0.537

Alkaline phosphatase/98–279 (U/Lit)	Before the intervention	399.32 ± 174.604	395.84 ± 220.363	328.81 ± 159.707	0.192
	After the intervention	364.15 ± 135.329	366.51 ± 190.718	322.00 ± 119.764	0.449

Platelet/140000–450000 (per microliter)	Before the intervention	174.95 ± 51.471	173.59 ± 59.048	189.08 ± 64.617	0.456
	After the intervention	179.47 ± 50.064	171.97 ± 65.012	178.58 ± 64.352	0.852

**Table 3:** Comparison of the pruritus severity between the groups.

Pruritus severity/groups	Medication (mean ± SD)	Placebo (mean ± SD)	Control (mean ± SD)	*P* value
Before the intervention	31.24 ± 6.95	30.05 ± 7.70	26.51 ± 7.08	**P**=0.017
Score change after the intervention	−17.24 ± 8.93	−5.91 ± 5.39	−3.43 ± 3.22	**P** < 0.001

**Table 4:** Comparison of the skin dryness severity between the groups.

Skin dryness/groups	Medication (mean ± SD)	Placebo (mean ± SD)	Control (mean ± SD)	*P* value
Before the intervention	2.27 ± 0.962	2.46 ± 1.01	2.16 ± 0.76	**P**=0.036
Score change after the intervention	−1.65 ± 0.91	−1.11 ± 0.79	−0.38 ± 0.54	**P** < 0.001

**Table 5:** Quality of life comparison (before and after intervention) in three groups.

Quality of life/groups	Medication (mean ± SD)	Placebo (mean ± SD)	Control (mean ± SD)	*P* value
Before the intervention	52.97 ± 11.68	51.54 ± 10.08	46.73 ± 6.91	*P*=0.018
After the intervention	8.29 ± 40	46.49 ± 9.66	44.81 ± 6.72	*P*=0.005
Score change after the intervention	−14.18 ± 7.89	−5.34 ± 4.50	−1.92 ± 2.86	*P* < 0.001

**Table 6:** Comparison of the sleep quality before and after the intervention in three groups.

Sleep quality/groups	Medication (mean ± SD)	Placebo (mean ± SD)	Control (mean ± SD)	*P* value
Before the intervention	3.22 ± 6.14	5.62 ± 3.59	5.49 ± 3.34	0.687
After the intervention	3.24 ± 2.41	4.54 ± 3.11	5.05 ± 3.21	**0.032**

**Table 7:** Fatty acid content of AG oil.

Fatty acid	Percentage
Linoleic acid	44.00
Oleic acid	20.99
Stearic acid	19.37
Palmitic acid	13.14
Arachidic acid	0.72
Behenic acid	0.67
11-Eicosenoic acid	0.46
Palmitoleic acid	0.30
Myristic acid	0.14

## Data Availability

The data that support the findings of this study are available on request from the corresponding author.
